# An evaluation of the comparative effectiveness of the RIPASA and Alvarado scoring systems in diagnosing acute appendicitis: a cross-sectional study

**DOI:** 10.1038/s41598-025-32589-4

**Published:** 2025-12-16

**Authors:** Madeeha Amanullah, Syed Raheel, Monika Devi, Sindhia Raj, Aroosa Khalil Ahmed, Amna Syed, Kifayatullah Dharejo, Muhammad Waqas Khan, Jemal Girma Mohammad

**Affiliations:** 1 Ghulam Muhammad Mahar Medical College and Teaching Hospital , Sukkur, Pakistan; 2 Jinnah Sindh Medical University , Karachi, Pakistan; 3 Dow University of Health Sciences , Karachi, Pakistan; 4 Tikur Anbessa Hospital , Addis Ababa, Ethiopia

**Keywords:** Acute appendicitis, Alvarado score, RIPASA score, Histopathology, Diseases, Gastroenterology, Health care, Medical research

## Abstract

The aim of this research is to evaluate the diagnostic precision of the Alvarado and RIPASA scoring systems in detecting acute appendicitis. This will be done by comparing their outcomes with histopathology, which is considered the most reliable benchmark for comparison. Accurate and prompt identification of acute appendicitis is crucial in order to avoid complications such as perforation and peritonitis. Over the years, several clinical scoring systems have been developed to aid in the diagnosis of acute appendicitis. The RIPASA (Raja Isteri Pengiran Anak Saleha Appendicitis) and Alvarado scoring systems are well recognised in the medical field. It is essential to do a comparison examination of these two scoring systems because to the possible changes in sensitivity, specificity, and overall diagnostic accuracy.This study included 145 male and female patients who presented with right iliac fossa discomfort and were thought to have acute appendicitis. Two diagnostic grading systems were used to guide surgical choices. Alvarado Score: Patients having a score of 7 or above were subjected to an appendectomy. RIPASA Score: Patients with a score less than 12 also had an appendectomy. 145 individuals included between the ages of 13 and 60, the Alvarado score was found to have a sensitivity of 34.4%, specificity of 75.8%, diagnostic accuracy of 51%, positive predictive value of 68.2%, and negative predictive value of 43.5% for diagnosing acute appendicitis. On the other hand, the RIPASA score performed better, with a sensitivity of 94.2%, specificity of 94.8%, diagnostic accuracy of 94%, positive predictive value of 96.5%, and negative predictive value of 91.6%. In the adult Pakistani population, the RIPASA scoring system demonstrates superior accuracy, sensitivity, specificity, PPV, and NPV compared to the Alvarado score, with a lower rate of negative appendectomies.

## Introduction

Acute appendicitis is a frequently occurring medical condition that requires immediate surgical intervention^1^. Since Reginald Fitz of Boston first established the clinical diagnosis of acute appendicitis in 1886 and recommended early appendectomy, the approach to diagnosis has largely relied on the patient’s medical history, physical examination, and a few laboratory tests^2–4^. Delaying surgery for acute appendicitis increases the risk of complications such as infection, abscess formation, and appendicular perforation, leading to higher morbidity and mortality^5,6^.

To improve diagnostic accuracy, several assessment schemes have been developed. The Alvarado scoring system, introduced in 1986, was designed in Western countries with distinct dietary and environmental conditions^6,7^. On the other hand, the Raja Isteri Pengiran Anak Saleha Appendicitis (RIPASA) scoring system was created by RIPAS Hospital in Brunei with the intention of being more suited for populations in Asia and the Middle East^8–10^. The Alvarado score has a sensitivity of 67.37% and specificity of 80%, while the RIPASA score exhibits a greater sensitivity of 94.74% but a little lower specificity of 60%^11,12^.

Acute appendicitis is a prevalent ailment, however even seasoned surgeons have challenges in accurately diagnosing it. This is especially true in young people, the elderly, and women who are of reproductive age, as many other gastrointestinal, genitourinary, and gynecological inflammatory conditions can also present with similar signs and symptoms^3,8^.

Diagnosing acute appendicitis remains challenging, particularly in young patients, the elderly, and women of reproductive age, due to the overlap in symptoms with other gastrointestinal, genitourinary, and gynecological conditions. While international studies have extensively evaluated the RIPASA and Alvarado scoring systems, local research is limited, leading to ongoing debate about their diagnostic accuracy in different populations. Both scoring methods are non-invasive and easy to compute, making them valuable tools in clinical practice to help reduce the incidence of unnecessary appendectomies.

## Materials and methods

A cross-sectional study conducted in accordance with the ethical principles outlined in the Declaration of Helsinki. Ethical approval was obtained from Institutional Ethical Committee of Ghulam Muhammad Mahar Medical College and teaching hospital Sukkur, Pakistan from May 1, 2022, to October 20, 2024. After obtaining ethics committee approval and informed consent, the study included 145 participants. The sample size of 145 participants was calculated using the WHO sample size calculator, based on an institutional negative appendectomy rate of 9.6% (from previous hospital records), 95% confidence interval, and 5% margin of error. Participants were selected using non-probability sequential sampling.

Decisions to perform appendectomy were made by senior consultants or registrars in general surgery, based on clinical judgment, lab and imaging findings, to standardize assessment across patients. The study included patients suspected of having acute appendicitis based on clinical evaluations, ultrasonography, and laboratory data, who subsequently underwent laparoscopic appendectomy.

Postoperative outcomes were recorded for all operated patients during hospitalization and follow-up visits. Conservatively managed patients were monitored for one month for complications or need for delayed surgery.

Both RIPASA and Alvarado scoring systems were applied to patients who met the inclusion criteria. The Alvarado score, consisting of 8 parameters, had a threshold of 7, while the RIPASA score, comprising 18 parameters.An Alvarado score of 7 or higher indicated the need for surgery, while the patients with Alvarado scores of 5–6 underwent further evaluation using the FACT (Focused Assessment of Clinical Tests) method to guide management and scores of 4 or lower ruled out appendicitis. RIPASA levels of 12 or more indicated the need for surgery, scores of 7.5–11.0 urged hospitalization and reevaluation, and scores of 7.0 or lower necessitated further evaluation. An examination of the appendix was performed to determine the existence of inflammation, and histology results confirmed acute inflammation.

### Inclusion and exclusion criteria

Patients aged ≥ 16 to ≤ 60 years who presented with suspected acute appendicitis and tenderness in the right iliac fossa (RIF) were included in the study. Patients were excluded if they were younger than 16 years or older than 60 years, pregnant, or had severe anemia (hemoglobin < 5 g/dL). Patients were excluded if conditions could interfere with accurate clinical assessment or scoring system evaluation, including skin pigmentation, appendicular mass, nephrolithiasis, generalized peritonitis, prior appendectomy, chronic abdominal pathology, recent abdominal surgery (< 90 days), or inguinal hernia. Furthermore, patients with a history of appendectomy, chronic abdominal pathology, abdominal surgery within the last 90 days, or an inguinal hernia were also excluded from the study.

### Data analysis procedure

Patients who underwent appendectomy had their tissue samples sent for histopathological analysis to confirm or refute the diagnosis of appendicitis. The histopathology results were then correlated with the scoring systems. Data were analyzed using SPSS version 26 (SPSS Inc.), with qualitative data presented through cross-tabulation and quantitative variables expressed as means and standard deviations. Chi-square tests were employed for analysis. Summary statistics included means, standard deviations, and proportions, while inferential statistics utilized the independent t-test, sensitivity, specificity with 95% confidence intervals (CI), and receiver operating characteristic (ROC) curve analysis, including the area under the curve.

## Results

The research included individuals aged 16 to 60 years, with an average age of 28.43 ± 6.96 years. The mean duration of symptoms was 3.06 ± 1.12 days, as shown in Table 1. The gender distribution of patients was 77.2% male and 22.8% female. Table 2 presents the diagnostic findings for acute appendicitis. The Alvarado score identified 44 patients (30.3%), the RIPASA score identified 85 patients (58.6%), and histopathology confirmed 87 cases (60%). The correlation between scoring systems and histopathology was statistically significant (*p* < 0.05). RIPASA showed higher sensitivity and specificity compared to Alvarado across all patient subgroups. The diagnostic performance of the Alvarado score demonstrated a sensitivity of 34.4%, specificity of 75.8%, and diagnostic accuracy of 51%. The positive predictive value (PPV) was 68.2%, and the negative predictive value (NPV) was 43.5%. In contrast, the RIPASA score showed significantly higher sensitivity (94.2%), specificity (94.8%), and diagnostic accuracy (94%). The PPV was 96.5%, and the NPV was 91.6% in diagnosing acute appendicitis, as presented in Table 4. Tables 3 and 4 display the stratified outcomes of Alvarado and RIPASA scores, as well as histological results, based on age, gender, and symptom duration.

**Table 1:** Demographics, gender distribution, and diagnostic findings in patients with suspected acute appendicitis (*n* = 145).

Category	Parameter	Value
Demographics	Age (years)	28.43 ± 6.96
	Duration of symptoms (days)	3.06 ± 1.12
Gender distribution	Male	112 (77.2%)
	Female	33 (22.8%)
	Total	**145 (100%)**
Diagnostic findings	Alvarado positive	44 (30.3%)
	Alvarado negative	101 (69.7%)
	RIPASA positive	85 (58.6%)
	RIPASA negative	60 (41.4%)
	Histopathology positive	87 (60%)
	Histopathology negative	58 (40%)
	Total cases	**145 (100%)**

Significant values are in bold.

**Table 2:** Statistical comparison of Alvarado and RIPASA scores against histopathology in diagnosing acute appendicitis.

Alvarado findings	Histopathology		Total
	Positive	Negative	
Positive	30 (TP)	14 (FP)	44
Negative	57 (FN)	44 (TN)	101
Total	87	58	145

RIPASA findings	Histopathology		Total
	Positive	Negative	
Positive	82 (TP)	3 (FP)	85
Negative	5 (FN)	55 (TN)	60
Total	87	58	145

**Table 3:** Statistical Comparison of Alvarado and RIPASA scores against histopathology in diagnosing acute appendicitis.

Comparison	Chi-square (χ²)	*p*-value	T-value	Interpretation
Alvarado vs. histopathology	1.31	0.253 (NS)	–	No significant association
RIPASA vs. histopathology	110.20	< 0.0001 (HS)	–	Strong significant association
Sensitivity (Alvarado vs. RIPASA)	–	–	−10.53	RIPASA has significantly higher sensitivity

*NS* Not significant, *HS* Highly significant.

**Table 4:** Comparative analysis of Alvarado and RIPASA scoring systems against histopathology for diagnosing acute appendicitis.

Variable	Alvarado positive	Alvarado negative	RIPASA positive	RIPASA negative	Histopathology positive	Histopathology negative	*P*-value	Sensitivity	Specificity	Diagnostic accuracy (DA)	PPV	NPV
Total cases (*n* = 145)	44 (30.3%)	101 (69.7%)	85 (58.6%)	60 (41.4%)	87 (60%)	58 (40%)	0.000	RIPASA: Higher Sensitivity	Alvarado: Higher Specificity	RIPASA: Better DA	RIPASA: Higher PPV	RIPASA: Higher NPV
Age 13–40 years (*n* = 130)	39 (TP)/77 (FP)	91 (FN)/53 (TN)	75 (TP)/77 (FP)	55 (FN)/53 (TN)	130	130	0.000	30%	40.7%	35%	33.6%	36.8%
Age 41–60 years (*n* = 15)	10 (TP)/10 (FP)	5 (FN)/5 (TN)	10 (TP)/10 (FP)	5 (FN)/5 (TN)	15	15	1.000	13.3%	33.3%	33%	33.3%	33.3%
Male (*n* = 112)	33 (TP)/70 (FP)	79 (FN)/42 (TN)	70 (TP)/70 (FP)	42 (FN)/42 (TN)	112	112	0.000	29.4%	37.5%	33%	32%	34%
Female (*n* = 33)	11 (TP)/17 (FP)	22 (FN)/16 (TN)	15 (TP)/17 (FP)	18 (FN)/16 (TN)	33	33	0.135	33.3%	48.4%	41%	39.3%	42.1%
Symptoms Duration ≤ 3 days (*n* = 101)	33 (TP)/59 (FP)	68 (FN)/42 (TN)	58 (TP)/59 (FP)	43 (FN)/42 (TN)	101	101	0.000	32.6%	41.5%	37%	35.9%	38.2%
Symptoms Duration > 3 days (*n* = 44)	11 (TP)/28 (FP)	33 (FN)/16 (TN)	27 (TP)/28 (FP)	17 (FN)/16 (TN)	44	44	0.000	25%	36.3%	31%	28.2%	32.6%

## Discussion

Aeute appendicitis is a prevalent surgical emergency globally, particularly among individuals under the age of 30, as reflected in this study where the mean patient age was 28.43 ± 6.96 years. Emergency appendectomies constitute approximately 10% of all abdominal surgeries performed in emergency departments^13–15^. While a surgeon’s clinical judgment is essential in diagnosing acute appendicitis (AA), relying solely on clinical assessment can lead to high rates of negative appendectomies, ranging from 15% to 30%^16,17^. Although contrast-enhanced computed tomography (CECT) provides high diagnostic accuracy, its use is limited in resource-constrained settings due to cost and availability^18^.

The Alvarado Score, introduced in 1986 by Alfredo Alvarado, remains a widely used clinical scoring system based on symptoms, signs, and laboratory parameters. It was based on a retrospective review of 305 patients with suspected appendicitis, identifying eight key criteria assigned diagnostic weights^19^. The Alvarado and Appendicitis Inflammatory Response (AIR) scores classify patients into low, intermediate, and high-risk groups, helping guide clinical decisions and prioritization of imaging. Among these, the AIR score is considered particularly effective in predicting AA^20^.

The Modified Alvarado Score (MAS), originally developed for pregnant women, was later adapted for general use due to its simplicity and accessibility. However, while MAS is commonly used in Western populations, it has demonstrated reduced diagnostic accuracy in Asian populations^21^. To address these limitations, the RIPASA score was developed at the Raja Isteri Pengiran Anak Saleha Hospital in Brunei, specifically for Asian populations. It accounts for ethnic and physiological differences and has shown greater diagnostic reliability than MAS^21-23^. RIPASA incorporates additional parameters such as age, gender, and duration of symptoms, which enhance its predictive ability in diverse Asian cohorts.

A study in Abbottabad showed that the Alvarado score reduced the negative appendectomy rate and had a specificity of 74.51%, PPV of 89.34%, NPV of 100%, and an overall accuracy of 91.88%. Based on these results, the study recommended routine application of the Alvarado scoring system in resource-limited settings^19^. Another study demonstrated that combining the Alvarado score with abdominal ultrasound further reduced the negative appendectomy rate to 5.1%, with a sensitivity of 94.62% and specificity of 87.80% at a cutoff of 6^23,24^.

Various scoring systems including Alvarado, MAS, Tzanakis, Ohmann, RIPASA, Pediatric Appendicitis, Lintula, and Fenyo-Lindberg have been developed to enhance diagnostic accuracy, but none has achieved universal acceptance^20,21,25^. A rural Indian study comparing three scoring systems identified RIPASA as the most effective for diagnosing complicated appendicitis^25^. With a cutoff of 5.75, it demonstrated AUC of 0.663 (*p* = 0.09), sensitivity of 90.7%, and specificity of 76.6%, supporting its utility in low-resource settings^20,28^.

Suhas Devanathan’s study reported only one false-positive result using the RIPASA score among patients undergoing emergency appendectomy, highlighting its high specificity^27^. Further studies by Chong et al, Regar MK et al and Alnajad et al. corroborated RIPASA’s diagnostic utility^21-23^. Dey et al. found a sensitivity of 94.2%, specificity of 70%, and a negative appendectomy rate (NAR) of 15.62%, which is comparable to our study’s NAR of 20%. These comparisons emphasize the consistency of RIPASA’s performance across different Asian populations.

In another Indian study, the NAR using RIPASA was just 2%, significantly lower than with MAS^21^. Across multiple studies, the RIPASA score’s NAR ranges from 0.7% to 17.39%, highlighting MAS’s limitations and supporting RIPASA’s superior diagnostic accuracy^20,21,24^. Our findings align with this body of evidence, demonstrating RIPASA’s superior sensitivity (94.2%) and specificity (94.8%) compared to the Alvarado score (sensitivity 34.4%, specificity 75.8%) in our cohort. Inteti et al. reported a NAR of just 2% with RIPASA, significantly lower than MAS (29). Across multiple studies, RIPASA achieved NARs from 0.7% to 17.39%, while MAS repeatedly underperformed^21-23,27-29^. Our findings mirror this pattern, with RIPASA showing higher sensitivity (94.2%) and specificity (94.8%) compared with Alvarado (34.4% and 75.8%)^30^.

The comparative analysis of RIPASA and Alvarado scores, as shown in Tables 5 and 6, confirms RIPASA’s superior sensitivity, specificity, and lower NAR. Table 1 shows RIPASA’s sensitivity across studies ranged from 73.63% to 100%, compared to Alvarado’s range of 34.4% to 82.42%. In most studies, RIPASA also had better or comparable specificity, with the highest reported at 94.8%. Table 2 emphasizes RIPASA’s effectiveness in reducing unnecessary surgeries, with studies such as Regar et al. (2017) and Devanathan et al. (2023) reporting NARs of 2.17% and 2%, respectively, markedly lower than the 10% reported for Alvarado in the same studies.

**Table 5:** Sensitivity and specificity of RIPASA and Alvarado scores.

Study author & year	Sample size	RIPASA sensitivity	RIPASA specificity	Alvarado sensitivity	Alvarado specificity
Alnjadat & Abdallah^9^	600	93.2%	-	73.7%	-
Nanjundaiah et al.^22^	206	96.2%	90.5%	58.9%	85.7%
Gopal et al.2016^1^	100	100%	11.11%	82.42%	44.44%
Regar et al.2017^12^	100	94.74%	60%	67.37%	80%
Goel et al., 2017^8^	100	95.6%	50%	63.3%	100%
Amanullah et al.^3^,	145	94.2%	94.8%	34.4%	75.8%
Mehbub et al.2023^23^	150	89.83%	59.38%	64.41%	53.12%
Devanathan et al.^19^	50	94.11%	93.75%	70.58%	68.75%
Inteti et al.2024^21^	110	73.63%	-	50.55%	-
Balakrishnan et al.^25^	76	90.7%	76.6%	57.4%	41.5%

These findings suggest that RIPASA is a more effective scoring system for diagnosing acute appendicitis, particularly in Asian populations for which it was specifically developed and validated. Our study adds further evidence supporting the clinical adoption of RIPASA in resource-limited settings, where advanced imaging may not be available.

**Table 6:** Negative appendectomy rate of RIPASA and Alvarado scores.

Study author & year	Sample size	RIPASA negative appendectomy rate	Alvarado negative appendectomy rate
Alnjadat & Abdallah, 2013^9^	600	7.8%	8%
Gopal et al.2016^1^	100	8.1%	6.3%
Regar et al.2017^12^	100	2.17%	1.54%
Mehbub et al.2023^23^	150	10.91%	16.48%
Devanathan et al.2023^19^	50	2%	10%

However, our study is limited by a relatively small sample size (145 patients) and a single-center design, which may limit the generalizability of the results. Additionally, excluding patients with comorbidities restricts the applicability of findings across more diverse clinical presentations. Despite these limitations, the study offers a valuable head-to-head comparison between RIPASA and Alvarado scores, reinforcing RIPASA’s diagnostic advantages in this population.

## Conclusion

The RIPASA scoring system demonstrates superior accuracy, sensitivity, specificity, positive predictive value (PPV), negative predictive value (NPV), and lower rates of negative appendectomy when used to the Pakistani population, in comparison to the Alvarado scoring system.

Particular benefits are provided to the public healthcare system in lower-middle-income countries such as Pakistan by the RIPASA grading system. Nevertheless, the utilization of this technology in our institutions is restricted by the absence of adequate research and clinical trials organized on a local scale. This study is a modest yet significant stride toward the establishment of the RIPASA score as a standard protocol for the diagnosis of appendicitis, as well as the increase in awareness among surgeons regarding its applicability and advantages.

## Data Availability

The datasets used and analysed during the current study available from the corresponding author on reasonable request.
